# Genetic Diversity and Spawning Patterns of Small Yellow Croaker (*Larimichthys polyactis*) in a Large-Scale Pooling System

**DOI:** 10.3390/biology15090734

**Published:** 2026-05-06

**Authors:** Eun Soo Noh, Chun Mae Dong, Songhee Choi, Hyo Sun Jung, Jungwook Park, In Joon Hwang, Jung-Ha Kang, Yong-Woon Ryu

**Affiliations:** 1 Biotechnology Research Division, National Institute of Fisheries Science, Busan 46083, Republic of Korea; laperm@korea.kr (E.S.N.); ehdcnsao@naver.com (C.M.D.); jhs3010@korea.kr (H.S.J.); jjuwoogi@korea.kr (J.P.); astraroth@korea.kr (I.J.H.); genetics@korea.kr (J.-H.K.); 2 Subtropical Fisheries Research Institute, National Institute of Fisheries Science, Jeju 63610, Republic of Korea; thdgml1126@naver.com

**Keywords:** *Larimichthys polyactis*, genetic diversity, microsatellite, parentage assignment, mass-spawning pooling system, multi-time sampling

## Abstract

Although mass-spawning pooling systems are widely used for small yellow croaker (*Larimichthys polyactis*) aquaculture, they often induce severe genetic bottlenecks driven by reproductive skew. This study evaluated cross-generational genetic diversity and spawning patterns to propose an optimal genetic management strategy. We analyzed 1049 adult broodstock and 950 juvenile offspring using nine microsatellite markers. To mitigate reproductive skew, fertilized eggs were collected via multi-time sampling (19 times) over a two-month spawning season and reared to the juvenile stage. Genetic diversity was highly conserved across generations, with expected heterozygosity maintained at 0.860 in the offspring. Parentage assignment succeeded for 96.2% of the offspring (914 individuals), revealing 802 unique families, of which 89.9% (721 families) were singletons. Also, 60.9% of the broodstock contributed to reproduction, exhibiting a right-skewed participation distribution. Importantly, comparisons with a short-term single-event collection control group demonstrated that our multi-time strategy effectively prevented drastic reductions in effective population size (*N*_e_). These patterns highlight the species asynchronous spawning physiology and confirm that the strategy approximates random mating with minimal genetic drift. We suggest this long-term, multi-time egg collection method as an effective protocol for the sustainable genetic management of multiple-spawning marine fish.

## Simple Summary

The small yellow croaker is a commercially valuable marine fish experiencing population decline. To support stable production, aquaculture is increasingly utilized. However, mass-spawning in large communal tanks frequently leads to reproductive skew, where a few dominant individuals produce the majority of the offspring. This reduces the genetic diversity of the farmed fish, making them vulnerable to diseases and environmental changes. This study investigated whether continuous collection of fertilized eggs throughout the two-month spawning season could mitigate this skew. Using genetic parentage analysis, we found that collecting eggs multiple times allowed 60.9% of the adult fish to successfully reproduce. By preventing a few dominant individuals from monopolizing reproduction, this method maintained a genetic diversity level comparable to wild population. Because these fish naturally spawn asynchronously, extending the egg collection period allows a wider range of individuals to contribute. Adopting this multi-time sampling method will help secure the genetic stability and sustainability of farmed populations.

## 1. Introduction

The small yellow croaker (*Larimichthys polyactis*), belonging to the family Sciaenidae in the order Perciformes, is an important fishery resource with high commercial value in Northeast Asian waters [1]. Recently, the wild population of this species has drastically declined due to climate change and overfishing [2]. As a result, the aquaculture industry has emerged as an important alternative to replace these resources and ensure a stable protein supply. For the sustainable development of the aquaculture industry, the mass production of artificial seeds with superior traits is essential. In this process, the implementation of a systematic breeding program to evaluate and manage the genetic health of aquaculture populations is strongly required [3].

Aquaculture populations are characterized by a closed breeding environment where a large number of offspring are produced from a limited number of broodstock. In such environments, if the reproductive participation rate of a few specific broodstock becomes excessively high, there is a severe risk of a genetic bottleneck or inbreeding [4]. A reduction in genetic diversity due to inbreeding causes severe inbreeding depression, such as decreased adaptability to environmental changes, increased malformation rates, and reduced disease resistance, which significantly hinders aquaculture productivity [5]. Efficient and long-term genetic management of elite populations requires continuous monitoring of the fluctuation patterns of genetic diversity between broodstock and offspring populations, and to establish scientific mating guidelines and rearing systems that can minimize the loss of genetic diversity [6].

Microsatellite markers are the most widely used tools worldwide for evaluating genetic diversity and performing parentage assignment in aquatic organisms [7]. Microsatellite markers exhibit a codominant mode of inheritance, possess a high mutation rate, and are evenly distributed across the genome, offering the advantage of high polymorphism [8]. Thanks to these characteristics, they provide excellent resolving power for analyzing the complex pedigree structures and clearly identifying parentage relationships of marine fish, which are often reared together in large numbers in massive tanks. Recent studies have also actively attempted to use these markers in various marine aquaculture species to evaluate the maintenance of genetic diversity across generations and verify genetic structures [9,10].

In the field of marine fish artificial seed production, a mass-spawning pooling system—where multiple male and female broodstock are housed in the same tank to induce natural spawning—is primarily adopted for the purpose of efficient mass production [11]. However, in this communal spawning method, reproductive skew, often referred to as Sweepstakes Reproductive Success (SRS), frequently occurs, where a few dominant individuals account for the majority of the total fertilized egg production [12,13]. Particularly for multiple spawners like the small yellow croaker, which lay eggs several times over the spawning season, if seeds are produced through only short-term, one-time egg collection, the number of broodstock participating in spawning is extremely limited, raising concerns about a rapid decline in genetic diversity [14]. Therefore, to maximize random mating under a pooling system, time-series multi-time sampling and long-term communal housing throughout the spawning period can be proposed as effective alternatives [15]. However, empirical genetic verification of long-term multi-time egg collection strategies in large-scale communal spawning systems remains extremely limited due to the practical difficulties of continuous long-term sampling and the methodological complexities of parentage assignment in mass-spawning population.

To explore efficient genetic management strategies for elite populations of small yellow croaker, we comparatively analyzed the genetic diversity and spawning patterns of a broodstock population reared in a mass-spawning pooling system and the offspring population produced from them over an extended period. By utilizing verified markers, we aimed to confirm whether genetic health is stably maintained across generations and quantitatively determine the effect of a long-term, multi-time sampling approach on inducing random mating and preventing specific family skew in communal spawning through large-scale parentage assignment. The results of this study will provide an optimal Standard Operating Procedure (SOP) for maximizing genetic diversity during artificial seed production and serve as crucial foundational data for designing sustainable breeding programs for aquatic genetic resources in the future.

## 2. Materials and Methods

### 2.1. Sample Collection

**Figure 1:**
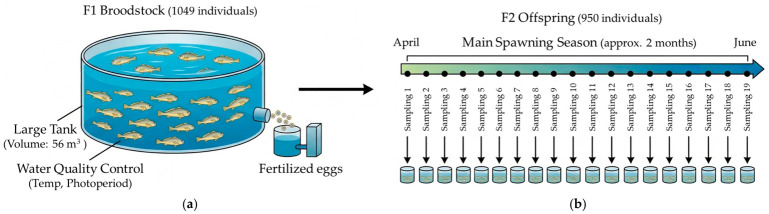
Schematic diagram of the large-scale pooling system (**a**) and time-series sampling strategy (**b**).

The small yellow croaker (*Larimichthys polyactis*) population used in this study was obtained from the Subtropical Fisheries Research Institute, National Institute of Fisheries Science (NIFS), Republic of Korea. For the efficient and long-term genetic management of the elite aquaculture population, a selected broodstock population (F1, 1049 individuals in total, sex ratio unknown) produced in 2023 with verified genetic health and maturity was established as the experimental group. This broodstock population was housed in a large tank using a mass-spawning pooling system to induce natural spawning and many-to-many random mating. Specifically, the broodstock (*n* = 1049) were maintained in a 56 m^3^ tank under a natural photoperiod and a constant water temperature of 21 °C. During each spawning event, approximately 5000 mL of fertilized eggs were collected. Subsequently, fertilized eggs produced from them during the main spawning season in 2025 were collected, hatched, and reared to the juvenile stage. To strictly prevent survival bias or over-representation of specific spawning events, the eggs collected from each of the 19 events were reared in 19 separate tanks under identical conditions. Once they reached the juvenile stage, 50 individuals were randomly sampled from each tank. The collected samples were promptly preserved in 99% ethanol for further genetic analysis. The resulting offspring population (F2, 950 individuals in total) was finally obtained and utilized for cross-generational genetic diversity fluctuation and parentage assignment analyses. To prevent reproductive skew towards specific broodstock that often occurs during communal spawning and to precisely analyze spawning characteristics, time-series multi-time sampling was conducted a total of 19 times over the approximately two-month main spawning season (from 9 April to 3 June). Since *L. polyactis* is a multiple-spawning species exhibiting asynchronous gonad development, the exact intervals between sampling events were uneven, as eggs were collected opportunistically whenever natural communal spawning occurred (Figure 1).

### 2.2. Genomic DNA Extraction

For genetic analysis, genomic DNA was extracted from the fin tissue of the obtained broodstock (F1, 2023) and the whole-body tissue of the offspring (F2, 2025) using the magnetic bead-based ARA MagNa Tissue DNA Isolation Kit (LAS, Gimpo, Republic of Korea). To ensure rapid and uniform processing of the large-scale samples totaling 1999 individuals, the KingFisher Flex system (Thermo Fisher Scientific, Waltham, MA, USA), an automated DNA extraction instrument, was utilized. As a specific pretreatment process, the collected tissue samples were mixed with 140 µL of Proteinase K buffer and 10 µL of Proteinase K (20 µg/mL) and then incubated overnight in a 56 °C incubator (JSR, Gongju, Republic of Korea) to completely lyse the tissues. The lysed sample solution was mixed with 650 µL of lysis buffer and 40 µL of magnetic beads, loaded into the automated equipment, and high-purity DNA was separated and purified in batches according to the manufacturer’s protocol. The concentration and purity of the isolated genomic DNA were measured using the NanoPhotometer N60 (Implen, Munich, Germany), after which the DNA was diluted to an appropriate concentration for analysis and stored in a −20 °C freezer to maintain stability.

### 2.3. PCR Amplification and Genotyping

For the genotyping and parentage assignment of the populations, a set of nine highly polymorphic, small yellow croaker-specific microsatellite (MS) markers, previously developed and verified, was utilized [10]. To enhance the precision of the polymorphism analysis, fluorescent dyes (6-FAM, HEX, TAMRA; Bionics, Daejeon, Republic of Korea) were labeled at the 5′ end of the forward primer of each marker pair. Multiplex PCR amplification was performed in a total reaction volume of 10 µL, comprising 1 µL of 10× Taq PCR buffer, 0.2 µL of 10 mM dNTP mixture, 0.1 µL of Hs Taq DNA polymerase (2.5 U/µL; TNT Research, Sejong, Republic of Korea), 0.4 µL each of 10 mM forward and reverse primers, and 1 µL of template DNA at a concentration of 30–50 ng, with the final volume adjusted using sterile distilled water. The reaction was conducted using a Veriti 96-well Thermal Cycler (Applied Biosystems, Foster City, CA, USA). The PCR cycling conditions consisted of an initial pre-incubation at 95 °C for 10 min, followed by a step-down thermal profile: denaturation at 94 °C for 50 s, annealing for 50 s (5 cycles at 59 °C, 5 cycles at 58 °C, and 32 cycles at 57 °C), and extension at 72 °C for 60 s, with a final full extension at 72 °C for 7 min. The presence of amplified products was initially confirmed through 1.8% agarose gel electrophoresis. Subsequently, the amplified fluorescent PCR products were diluted at a ratio of 1:10 to 1:50, and 1 µL of the dilution was added to 10 µL of a mixture containing Hi-Di formamide and the internal size standard GeneScan 400HD ROX (Applied Biosystems, Foster City, CA, USA) in an 8:2 ratio. This mixture was denatured at 95 °C for 2 min, rapidly chilled to 4 °C, and then subjected to capillary electrophoresis using an ABI 3730XL Genetic Analyzer (Applied Biosystems, Foster City, CA, USA). The resulting fluorescent peak data were analyzed using GeneMapper version 4.0 software (Applied Biosystems, Foster City, CA, USA) to read the allele sizes of each individual and determine the final genotypes.

### 2.4. Statistical Analysis of Genetic Diversity and Structure

To evaluate the genetic diversity and differentiation of the small yellow croaker populations, statistical analyses were performed using GenAlEx version 6.5 and Arlequin version 3.5 [16,17]. Basic genetic diversity indices, including the total number of alleles (*N*_a_), number of effective alleles (*N*_ea_), and Shannon’s information index (*I*), were calculated using GenAlEx. The observed heterozygosity (*H*_o_), expected heterozygosity (*H*_e_), deviation from Hardy–Weinberg equilibrium (HWE), and the inbreeding coefficient (*F*_IS_) for each locus and population were calculated using Arlequin. Furthermore, to assess the degree of genetic structure and differentiation between the broodstock and offspring populations, pairwise F_ST_ values were estimated using Arlequin, with statistical significance tested through 10,000 random permutations.

Additionally, the effective population size (*N*_e_) of the breeding population was estimated using the temporal changes in allele frequencies between the F1 and F2 generation via NeEstimator v2.1 (Jorde and Ryman estimator, *P*_crit_ = 0.02). Statistical differences in genetic diversity parameters (*H*_o_, *H*_e_, *N*_a_) between the F1 broodstock and F2 offspring across the 9 loci were evaluated using a Wilcoxon signed-rank test, with a significant level set at *p* < 0.05. To quantitatively evaluate the genetic benefits of the multi-time sampling strategy, we analyzed a subset of offspring (sampled on March 3, *n* = 100) derived from a single mass-spawning event to represent a conventional short-term egg collection practice. The *N*_e_ of this short-term control cohort was calculated and directly compared with that of the total multi-time sampling cohort (*n* = 950).

### 2.5. Parentage Assignment

To analyze the reproductive contribution of the broodstock that participated in spawning and the detailed pedigree structure of the 950 offspring (F2), Cervus 3.0.7, a genotype-based parentage assignment program, was utilized [18]. Simulations were conducted beforehand based on the allele frequencies of the 9 established markers. In the simulation parameters, the proportion of loci typed was 0.9989, the proportion of loci mistyped was set to 0.01, and the error rate in likelihood calculations was also set to 0.01. Because clear morphological sex identification of individual live broodstock is practically impossible without causing extreme stress or mortality, exact sex ratios could not be determined. Consequently, parentage assignment was performed using the ‘sexes unknown’ module. By applying the calculated Logarithm of the Odds (LOD) scores and Delta values, parent pairs were assigned at a 95% confidence level. Through the derived parentage matching results, the broodstock participation frequency across the 19 sampling events was calculated, and the occurrence of reproductive skewness towards specific individuals and the success of random mating within the mass-spawning pooling system were comprehensively evaluated.

## 3. Results

### 3.1. Cross-Generational Genetic Diversity

Cross-generational changes in genetic diversity were quantified using nine microsatellite markers in the elite broodstock population (F1, *n* = 1049) and their communally reared offspring (F2, *n* = 950) (Table 1). The analysis revealed that the mean total number of alleles (*N*_a_) slightly decreased from 17.222 (±1.516) in the broodstock to 16.667 (±1.269) in the offspring. This was due to the inevitable natural loss of rare private alleles with extremely low occurrence frequencies within the population (mean 1.000 → 0.444). However, the number of alleles with a frequency of ≥ 5% (*N*_a_ Freq. ≥ 5%), which constitutes the substantial genetic framework of the population, remained exactly identical across generations at 6.778 in both populations. In addition, the number of effective alleles (*N*_ea_), a key indicator of the population’s evolutionary adaptive potential, showed a significant and positive upward trend from 7.075 to 7.290, and Shannon’s information index (*I*) increased from 2.222 to 2.239.

The expected heterozygosity (*H*_e_), which represents the overall genetic health of the population, was highly maintained without statistical significance between the broodstock (0.856 ± 0.021) and offspring (0.860 ± 0.022) populations (Wilcoxon signed-rank test, *p* = 0.301). Similarly, the observed heterozygosity (*H*_o_) slightly increased from 0.780 (±0.085) in the broodstock to 0.795 (±0.089) in the offspring, but this difference was also not statistically significant (*p* = 0.129). Notably, while the broodstock population showed significant deviations from Hardy–Weinberg equilibrium (HWE) across all 9 loci, the offspring population recovered equilibrium in a majority of the loci (5 out of 9 loci: Locus 4, 5, 7, 8, 9; *p* > 0.05), strongly indicating the successful induction of random mating. Consequently, the inbreeding coefficient (*F*_IS_) decreased from 0.089 in the broodstock to 0.075 in the offspring, confirming that significant inbreeding depression was effectively prevented within the mass-spawning pooling system. Additionally, the pairwise fixation index (*F*_ST_), indicating the degree of genetic differentiation between populations, recorded an extremely low value (*F*_ST_ < 0.01), confirming that genetic drift did not occur during the generational transition.

Based on the temporal method, the *N*_e_ of the total multi-time sampling cohort (19 event, sampled from 9 April to 3 June; *n* = 950) was estimated at 188.3 (95% CI: 181.1–246.1). However, the analysis of the separate short-term control group (sampled on 3 March; *n* = 100) revealed a drastic reduction in genetic health, with the *N*_e_ plummeting to 24.9 (95% CI: 18.2–32.7) (Table 2).

### 3.2. Marker Polymorphism and Resolving Power

Verification of marker efficiency based on the genotype data of all 1999 individuals yielded a mean Polymorphism Information Content (PIC) of 0.8443, confirming that all 9 applied markers possessed extremely high locus variability (Table 3). Additionally, the estimated null allele frequencies (*F*_null_) calculated by CERVUS across the 9 loci ranged from −0.0078 to 0.1391. Under the challenging analytical condition of ‘sexes unknown’ necessitated by the difficulty of clear morphological sex identification of the individual broodstock, the combined non-exclusion probability for a parent pair was calculated as 2.003 × 10^−9^. This represents an error probability of less than one in a billion, proving that this marker set has exceptional statistical resolving power capable of establishing parent–offspring relationships with high precision and without false-positive matching, even in large-scale aquaculture populations.

### 3.3. Parentage Assignment and Pedigree Structure

**Figure 2:**
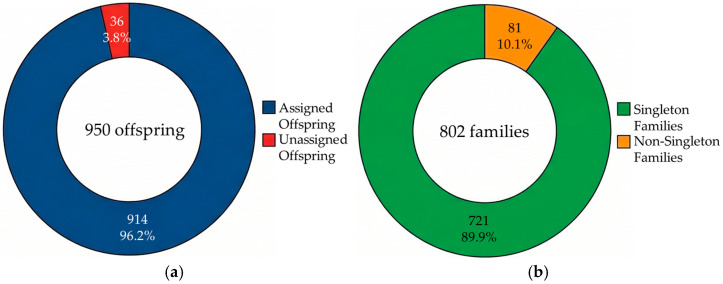
Pedigree structure (**a**) and proportion of singleton families (**b**).

Based on the proven high resolving power, Cervus simulations were performed, resulting in the successful assignment of parent pairs for all 914 offspring (96.2%) at a strict 95% confidence level. A fine-scale pedigree structure analysis of the offspring population based on the derived parentage matching data revealed that a total of 802 unique families were formed within the cohort. Of these 802 families, 721 (89.9%) were identified as “singleton” families that produced only a single offspring (Figure 2). The occurrence frequency of a few dominant families producing multiple offspring was extremely low, suggesting that reproductive contribution was evenly and broadly distributed across the effective broodstock within the large tank.

### 3.4. Spawning Frequency and Asynchronous Patterns

**Figure 3:**
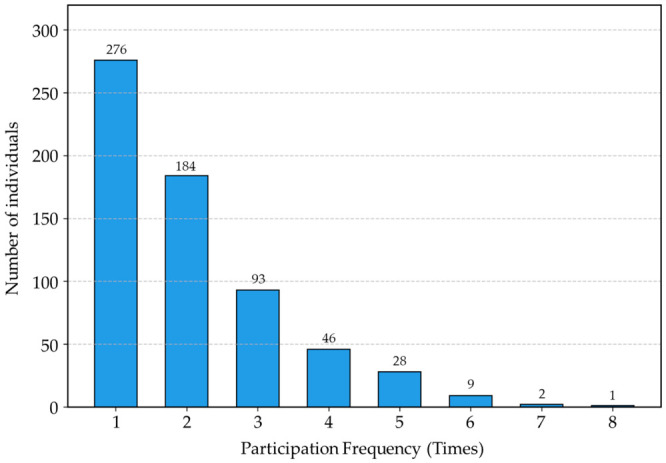
Spawning participation frequency among the *L. polyactis* broodstock.

**Figure 4:**
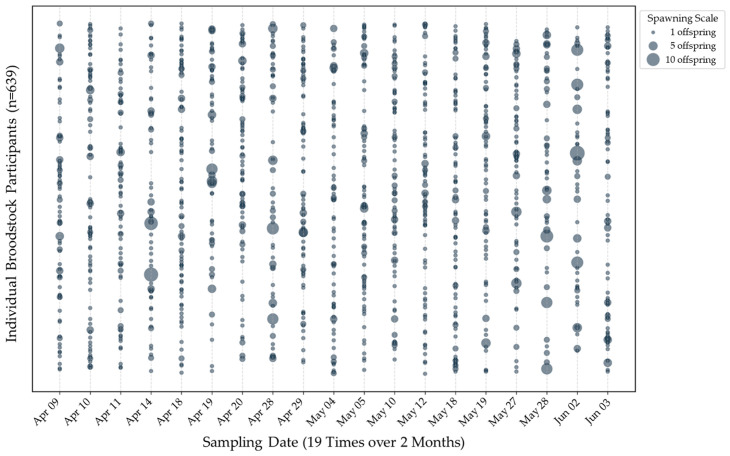
Asynchronous reproductive patterns across 19 sampling times (*n* = 50 per event).

Through time-series multi-time sampling conducted 19 times over the main spawning season of approximately two months (April–June), the spawning participation frequency and timing of each individual were precisely analyzed. Among the 1049 housed broodstock, 639 individuals (60.9%) contributed to offspring production at least once. Quantifying the distribution by participation frequency showed that broodstock participating only once held the highest share with 276 individuals (43.2%). This was followed by 2 times (184 individuals), 3 times (93 individuals), 4 times (46 individuals), and 5 times (28 individuals), clearly demonstrating a highly right-skewed distribution curve where the number of individuals exponentially decreases as the participation frequency increases (only 1 individual participated a maximum of 8 times) (Figure 3). Furthermore, a cross-analysis of daily spawning patterns revealed that rather than engaging in synchronous spawning, the broodstock clearly exhibited a highly asynchronous spawning physiology, participating intermittently with intervals of days to weeks between individuals (Figure 4).

## 4. Discussion

The preservation of genetic diversity across generations (*H*_e_ 0.856 → 0.860; Wilcoxon signed-rank test, *p* = 0.301) observed in this study sharply contrasts with previous findings reported for other marine aquaculture populations. Generally, a decrease in the number of effective alleles (*N*_ea_) and loss of diversity are commonly reported during the artificial seed production of other marine fish [19]. While previous studies have shown that the observed and expected heterozygosity (*H*_o_ and *H*_e_) of wild small yellow croaker populations are maintained at extremely high levels of 0.762–0.799 and 0.845–0.858, respectively [9]. In contrast, standard cultured populations of closely related marine species, such as the large yellow croaker (*Larimichthys crocea*), frequently experience a sharp drop in both indices (*H*_o_, 0.570–0.647; *H*_e_, 0.591–0.649) due to severe domestication bottlenecks [14]. However, the F2 offspring population in this study exhibited a *H*_o_ of 0.795 and an *H*_e_ of 0.860, which closely rival wild populations, empirically demonstrating that this rearing system can effectively mitigate genetic bottlenecks, prevent inbreeding depression, and substantially maintain the genetic integrity of a core nucleus breeding population.

In our study, the F1 broodstock exhibited significant deviations from Hardy–Weinberg Equilibrium (HWE) across all 9 loci. This deviation is likely attributed to the Wahlund effect, as the F1 cohort was originally established by pooling individuals from multiple temporal spawning event, effectively mixing sub-populations with slightly different allele frequencies [20,21]. This interpretation is empirically supported by the F2 offspring data; following natural communal spawning, HWE was restored in a majority of the loci (5 out of 9), demonstrating that the initial deviations were temporary consequences of population mixing rather than inherent marker flaws. Furthermore, the potential presence of low-frequency null alleles, a common occurrence in the highly polymorphic microsatellites of marine fishes, may have partially contributed to the heterozygote deficiency [22,23]. Based on CERVUS allele frequency analysis, the estimated null allele frequencies (*F*_null_) across the 9 loci ranged from −0.0078 to 0.1391. While two loci exhibited moderate frequencies, they remained well within acceptable limits. Consequently, the sufficient resolving power of the marker panel and the likelihood-based algorithm of CERVUS minimize the impact of these deviations on the accuracy of the parentage assignment.

In studies of other mass-reared marine fish, Sweepstakes Reproductive Success (SRS) is highly prevalent, a phenomenon characterized by a severe reproductive skew where a small minority of dominant broodstock monopolizes the vast majority of offspring production [24]. Despite using only nine loci, the extremely high PIC values and negligible non-exclusion probability ensured robust parentage assignment accuracy in our analysis. Based on this robust analytical power, this study showed that 60.9% of the broodstock contributed, 89.9% of the offspring families were singletons, and the participation followed a highly right-skewed distribution. This is clearly distinct from the severe monopolistic spawning patterns of other species and confirms that the long-term fertilized egg collection method effectively mitigated the SRS phenomenon. Additionally, while 3.8% (36/950) of the offspring remained unassigned, this negligible proportion is inherently expected under the ‘sexes unknown’ assignment module. The exponential increase in pairwise comparisons requires extremely strict statistical thresholds to achieve 95% confidence, which occasionally leaves some true parent pairs unresolved [25]. Therefore, this minor unassigned fraction does not represent outside contributions and does not bias the overall highly diverse family structure.

As empirically demonstrated by the participation frequency (Figure 3), individual broodstock spawned between 1 and 8 times over the two-month period. This reflects the species’ multiple-spawning rhythm and the lack of gonad development synchrony among individuals. Consequently, this asynchronous physiology elucidates the vulnerability of mass-spawning systems to SRS. Under conventional short-term egg collection, family structures often become highly skewed because only a limited fraction of broodstock synchronized to spawn within a narrow window can contribute to the next generation. Currently, many aquaculture sites maintain the practice of intensive egg collection for only 1 to 2 days during the peak spawning season. However, this leads to a significant reduction in the effective population size (*N*_e_) by amplifying only the genes of a very small number of individuals that happened to be reproductively active at that time [26]. To empirically evaluate this, we analyzed a separate short-term control group consisting of 100 offspring sampled from a single-time egg collection. The *N*_e_ of this short-term control cohort decreased significantly to 24.9, which is below the generally accepted safety threshold (*N*_e_ > 50) to prevent short-term inbreeding depression in aquaculture [27]. This physical evidence strongly indicates that conventional short-term practices are highly susceptible to severe SRS. Conversely, the *N*_e_ (188.3) achieved by our 19-time sampling demonstrates that continuous egg collection effectively mitigates this risk.

While this study provides robust empirical evidence for the benefits of multi-time sampling, several limitations must be acknowledged. First, the analysis is based on a single breeding season and cohort, requiring multi-year replicates to validate long-term consistency. Second, because parentage was assigned at the juvenile stage, potential survival selection during early developmental stages cannot be entirely ruled out, although our balanced sampling mathematically minimized this bias. Therefore, we strongly suggest this continuous multi-time sampling strategy as a highly effective genetic management protocol for *L. polyactis*. However, its broad generalization to species with different reproductive phenologies requires cautious, species-specific validation. Future studies should evaluate this strategy across multiple generations and test its applicability to other commercial sciaenids.

## 5. Conclusions

This study provides empirical molecular genetic evidence that long-term, multi-time sampling within a mass-spawning pooling system can successfully prevent genetic bottlenecks and maintain high genetic diversity in the artificial seed production of the small yellow croaker. By tracking 19 sampling events over a two-month period, we identified that the small yellow croaker exhibits highly asynchronous spawning behavior. This suggests that the conventional practice of short-term, intensive egg collection over just one or two days poses significant limitations for multiple spawners, as it inevitably triggers Sweepstakes Reproductive Success (SRS) and drastically reduces the effective population size.

Our findings demonstrate that pooling fertilized eggs continuously over the entire spawning season allows for a broad and even distribution of reproductive contributions across the broodstock, resulting in a predominant proportion of singleton families (89.9%), suppressed inbreeding (*F*_IS_ = 0.075), and high observed and expected heterozygosities (*H*_o_ = 0.795, *H*_e_ = 0.860) that are highly comparable to wild populations. To ensure the evolutionary adaptive potential and productivity of elite aquaculture populations, the time-series multi-time sampling strategy must be actively adopted as an essential Standard Operating Procedure (SOP). The genetic baseline and parentage assignment protocols established in this study will serve as a fundamental cornerstone for designing sustainable, pedigree-based selective breeding programs for marine aquatic resources. As these findings are based on a single breeding season and cohort of *L. polyactis*, further multi-year replications will be valuable to confirm the long-term consistency of this strategy and its broader applicability to other marine species.

## Figures and Tables

**Table 1:** Genetic diversity of the broodstock (F1) and offspring (F2) populations of small yellow croaker in a mass-spawning pooling system.

Population	*N*	*N*_a_	*N*_a_ (Freq. ≥ 5%)	*N*_ea_	*I*	*H*_o_	*H*_e_	*F*_IS_	HWE (*p* > 0.05)
Broodstock (F1)	1049	17.222 (±1.516)	6.778	7.075	2.222	0.780	0.856	0.089	0/9 loci
Offspring (F2)	950	16.667 (±1.269)	6.778	7.290	2.239	0.795	0.860	0.075	5/9 loci

**Table 2:** Comparison of effective population size (*N*_e_) between long-term multi-time sampling (*n* = 950) and short-term control cohort (single event, *n* = 100).

Sampling Strategy	Lowest Allele Frequency	Estimated *N*_e_	95% Confidence Interval (Parametric)
Multi-time sampling (19 event, *n* = 950)	0.05	165.9	112.4–229.8
	0.02	188.3	138.1–246.1
Short-term sampling (1 event, *n* = 100)	0.05	26.8	18.0–37.5
	0.02	24.9	18.2–32.7

**Table 3:** Summary of marker polymorphism and parentage assignment.

Parameter	Value
Number of loci	9
Total number of genotyped individuals	1999
Mean Polymorphism Information Content	0.8443
Combined non-exclusion probability (parent pair)	2.003 × 10^−9^
Parentage assignment module	Sexes unknown
Assignment confidence level	95% (Strict)
Parentage assignment success rate	96.2% (914/950)
Total assign unique families	802
Proportion of singleton families	89.9% (721/802)

## Data Availability

The data sets generated and/or analyzed in the present study are available from the corresponding author upon reasonable request.
